# Occurrence of *bla*_NDM_ Variants Among Enterobacteriaceae From a Neonatal Intensive Care Unit in a Northern India Hospital

**DOI:** 10.3389/fmicb.2018.00407

**Published:** 2018-03-07

**Authors:** Nayeem Ahmad, Shamsi Khalid, Syed M. Ali, Asad U. Khan

**Affiliations:** ^1^Interdisciplinary Biotechnology Unit, Medical Microbiology and Molecular Biology Lab, Aligarh Muslim University, Aligarh, India; ^2^Pediatrics Department, Jawaharlal Nehru Medical College, Aligarh Muslim University, Aligarh, India

**Keywords:** NDM, carbapenemase, Hospital, NICU, ERIC-PCR, antibiotic resistance, enterobacteriaceae

## Abstract

Carbapenem-resistance among enterobacteriaceae has become a global health concern. The objective of this study was to understand NDM producing enterobacteriaceae and their genetic basis of resistance, spreading in neonatal intensive care unit. Carbapenem resistant NDM producing enterobacteriaceae isolates were recovered from rectal swab and blood sample of infants admitted in NICU. These were determined by using Carba-NP test. All isolates were identified using BD Phoenix^TM−100^ and MICs were determined by broth microdilution method. The *bla*_NDM_ and associated resistant markers were checked by PCR followed by sequencing. Moreover, ERIC-PCR and genetic environment of *bla*_NDM_ gene were also performed for the analysis of clonal relationship and genetic surrounding of the strains. We characterized 44 isolates with *bla*_NDM_ variants in *Escherichia coli* (45.5%), *Klebsiella pneumoniae* (40.9%), *Citrobacter freundii* (4.5%), *Citrobacter braakii* (2.3%), *Klebsiella oxytoca* (2.3%), *Enterobacter cloacae* (2.3%), *Enterobacter aerogenes* (2.2%) from NICU, showing resistance against all antibiotics except colistin and polymixin B. IS*Aba125* and bleomycin gene were found surrounding all *bla*_NDM_ variants, besides class I integron on plasmid. (ERIC)-PCR data revealed non-clonal relatedness among most of the isolates. The transfer of resistant markers was confirmed by conjugation experiment. The PCR-based replicon typing was carried out using DNA of transconjugants. These isolates carried NDM-1 (20.45%), NDM-4 (36.36%), NDM-5 (38.64%), NDM-7 (4.55%), along with OXA, CMY, and SHV variants on conjugative plasmid of IncFIA, IncFIC, IncF, IncK, IncFIB, IncB/O, IncHI1, IncP, IncY, IncFIIA, IncI1, and IncN types. An increased number of carbapenem-resistant NDM producing enterobacteriaceae isolates recovered from NICU which is alarming signal for health workers and policy makers. Hence, it is utmost important to think about infection control measures.

## Introduction

Emergence of New-Delhi Metallo-β-lactamase (NDM) producers is a matter of concern. The spread of MBL-producing enterobacteriaceae has increased from 2008 onward with the discovery of an ST14 *Klebsiella pneumoniae* with a new MBL gene, *bla*_NDM−1_, from a 59-years old Swedish patient who received healthcare in New Delhi, India (Yong et al., 2009). Indian subcontinent are the most endemic region for the spread of NDM-type MBLs and prevalence rates of NDM-producing enterobacteriaceae were found in range of 5–18.5% in Indian and Pakistan hospitals (Perry et al., 2011; Bharadwaj et al., 2012). In other regions (except the Balkan and Middle East countries), NDM-type MBLs are described mostly as periodic occurrences (Dortet et al., 2014). Carbapenem-resistant microorganisms have become an alarming phenomenon in children (Logan, 2012). A recently published study in USA reported that the frequency of carbapenem resistance increased from 0% in 1999–2000 to 0.47% in 2010–2011 among Enterobacteriaceae isolates in children (Logan et al., 2015). To date, 19 variants of NDM-type carbapenemases (NDM-1 to NDM-19) have been identified (http://www.lahey.org/Studies/other.asp#table1). These variants were identified in expanded species of Gram-negative bacteria and were found to have variation either by multiple residues at different positions or by replacing single amino acid. Recently, an NDM-4, NDM-5, and NDM-7 producing *Enterobacter aerogenes* from NICU of Indian hospital were reported by our group (Ahmad et al., 2017a). The most widespread variants were found in Indian sub-continent, are NDM-1, NDM-4, NDM-5, NDM-6, and NDM-7 (Khan et al., 2017). Whereas, several types of carbapenemases, such as KPC, IMP, OXA-48, VIM, and New Delhi metallo-β-lactamase (NDM), have been identified globally (Pitout et al., 2015; Logan and Weinstein, 2017).

NDM producing bacteria are resistant to almost all antibiotics, except polymyxins (Kumarasamy et al., 2010). But, the hope of colistin and polymyxins as treatment option has become limited after the discovery of MCR-1 gene in human and animals (Liu et al., 2016). The indiscriminate nature of the gene encoding NDM-1 has made major problem in neonatal intensive care units (NICU). In NICU, high consumption of antimicrobial agents, numerous indwelling devices, and staff rotativity, may further complicate the problem (Zaidi et al., 2005).

In enterobacteriaceae, *bla*_NDM−1_ is generally located on conjugative plasmids, ranging from 50 to 200 kb in size and belongs to several incompatibility groups, such as IncL/M, IncHI1, IncFIIs, IncF, or untypable, enabling transfer, and rapid dissemination of multidrug resistance (Poirel et al., 2011).

Our study was designed to evaluate retrospectively the spread of NDM producing Enterobacteriaceae and their genetic basis in neonatal intensive care unit of one of the north Indian tertiary care hospital.

## Materials and methods

### Collection of bacterial strains and hospital setting

A total of 750 Enterobacteriaceae clinical isolates were screened from blood and rectal swab of 1,140 neonates admitted in neonatal intensive care unit (NICU) of Jawaharlal Nehru Medical College and Hospital (JNMCH), Aligarh Muslim University, Aligarh, India, during the period, December 2015 to January 2017 in which 308 isolates were found to be carbapenem resistant. It is a tertiary care hospital of 1,300 bed capacity, in which 90 beds were allotted for pediatric patients and 35 beds for the NICU. Patients enrolled in the study were those who enrolled in the active surveillance system (NICU stay 48 h and weekly surveillance swabs taken at least once). Neonates admitted to the ward before December 2015 and/or discharged after January 2017, were excluded.

### Ethical approval

A formal consent from the institutional ethical committee was taken and clearance was obtained from the institute's ethics committee. Participants/guardians had provided written, informed consent to participate in the study. We have a specific format to get the consents of patients/ parents of minors. These formats were made according to the Institutional ethics committee's guidelines. These forms are confidential and cannot be disclosed as per the guide lines. Institutional ethical committee has already approved. The name of committee/board is “Institutional Ethical Committee of Interdisciplinary Biotechnology Unit [Biot/307/01.06.13],” Aligarh Muslim University, Aligarh, India.

### Antimicrobial susceptibility, metallo-β-lactamase (MBL), and MICs testing

Antimicrobial susceptibility was determined by the standard disc diffusion method using Mueller Hinton agar plate as per the Clinical and Laboratory Standards Institute guidelines (CLSI, 2016). More than 05 colonies were picked from MH agar plate for antimicrobial susceptibility testing and MBL detection. Detection of metallo-β-lactamase activity was performed, using two imepenem discs (10 μg), one containing 10 μl of 0.1M anhydrous Ethylene Diamine Tetra-Acetic Acid (EDTA). The discs were placed 25 mm distance (center to center) on Mueller-Hinton agar plates (Ahmad et al., 2017b). Minimum Inhibitory Concentrations (MICs) for antimicrobial agents were determined using broth micro dilution method, according to the guidelines of the CLSI.

### Carba NP test for detection of carbapenemase

Carba NP test is a biochemical method used for the detection of carbapenemase activity in enterobacteriaceae isolates, performed as described earlier (Nordmann et al., 2012).

### Isolate identification

The species level identification of isolates were performed by using BD Phoenix^TM−100^ automated microbiology system using panel NMIC/ID-55 (Gram negative susceptibility card) and further validated by 16s rRNA sequencing using primer as described previously (Shemesh et al., 2012).

### Polymerase chain reaction (PCR) amplification and sequence analysis

PCR (Applied Biosystems model-9902 Verity thermo cycler) amplification was performed using primers as described previously (Poirel et al., 2011; Ali et al., 2014) for *bla*_NDM_ and other resistant marker (*bla*_VIM_, *bla*_OXA−1_, *bla*_OXA−9_, *bla*_CMY_, *bla*_TEM_, *bla*_SHV_, and *bla*_KPC_). Amplicons of NDM were purified from the gel using gel extraction kit (Thermo Fisher Scientific), following manufacturers' protocol and then sequenced for DNA sequencing at Sci Genom Labs Private Ltd, Cochin, India. The nucleotide and deduced protein sequences were analyzed with software available at the National Centre for Biotechnology Information Website (www.ncbi.nlm.nih.gov).

### Molecular characterization of plasmid

Plasmid DNA extraction and molecular size of multiple plasmids were identified by Kieser method (Kieser, 1984). Plasmid incompatibility group was determined by a PCR-based replicon typing (PBRT) method. Plasmid DNA was amplified by five multiplex and three simplex PCRs using 18 pair of primers as reported previously (Carattoli et al., 2005) that are recognized as Inc. replicon types: FIA, FIB, FIC, HI1, HI2, I1-Ic, L/M, N, P, W, T, A/C, K, B/O, X, Y, F, and FIIA.

### Conjugation experiment

The transfer of resistant markers (*bla*_NDM_, *bla*_CMY_, *bla*_OXA_, and *bla*_SHV_) was determined by conjugation, using an azide-resistant *E. coli* J53 strain as the recipient and isolates as donor (Walsh et al., 2011). Transconjugants were screened on Luria-Bertani agar supplemented with ceftazidime (10 μg ml^−1^) (Sigma-Aldrich) and sodium azide (100 μg ml^−1^) (HiMedia Laboratories, India). The PCR amplification confirmed the transconjugants having resistant markers.

### Genetic environment analysis

It was performed to identify the genes present at upstream and downstream of *bla*_NDM_ variants as described previously (Poirel et al., 2011).

### Integron analysis

The transconjugants of all the isolates, with *bla*_NDM_, were subjected to undergo integron analysis, using PCR amplification of 3′/5′ conserved segment along with *Int*1 and *Sul*1 as reported earlier (Dortet et al., 2012).

### Molecular genotyping of isolates

The clonally relatedness between NDM producing isolates were investigated by enterobacterial repetitive intergenic consensus-PCR (ERIC-PCR) using the primers ERIC-Forward (5′ATGT AAGCTCCTGGGGATTAAC-3′) and ERIC-Reverse (5′AAGTAAGGACTGGGGTGAGCG-3′), was performed as described earlier (Versalovic et al., 1991). Bio-Red Gel Doc system was used to scan gel image and analyzed the bands by PyElph version 1.4 Software to generate a dendrogram by the un weighted pair group method using arithmetic averages (UPGMA) clustering (PyElph) (Pavel and Vasile, 2012).

## Results

### Isolate identification

Of 44 isolates, *Escherichia coli* (*n* = 20; 45.5%), *K. pneumoniae* (*n* = 18; 40.9%), *Citrobacter freundii* (*n* = 2; 4.5%), *Citrobacter braakii* (*n* = 1; 2.3%), *Klebsiella oxytoca* (*n* = 1; 2.3%), *Enterobacter cloacae* (*n* = 1; 2.3%), *E. aerogenes* (*n* = 1; 2.2%), were identified.

### Antimicrobial susceptibility, metallo-β-lactamase (MBL), and MICs

Of 750 isolates, 44 were found to be New-Delhi Metallo-β-lactamase (NDM) producing enterobacteriaceae strains. All NDM producing strains were found highly resistant antibiotics, including carbapenems (imipenem and meropenem), cephamycin (cefoxitin), extended-spectrum cephalosporins (ceftazidime and cefotaxime), aminoglycoside (gentamicin and amikacin), monobactam (aztreonam), tetracycline (minocycline and tigecycline), fluoroquinolone (ciprofloxacin), except polymyxin and colistin. Metallo-β-lactamase (MBL) activity was present in all 44 NDM producing enterobacteriaceae isolates (Table 1). MICs data revealed high values against all tested antibiotics which were found in the range of 128 ≥ 4,096 μg ml^−1^ (Supplementary Table S1).

**Table 1:** Phenotypic and Genotypic Characterization of (NDM) producing enterobacteriaceae isolates from NICU setting.

**S.No**.	**Organism name**	**Isolate Id**	**Accession no**.	**NDM variant**	**Carba NP result**	**Metallo-β-lactamase**	Associated resistance markers^*^	No. of plasmid/Molecular size in kb^*^	Plasmid type^*^	Integron^*^	**Genetic environment of *bla*_NDM_**	
											**IS*Aba125***	***ble*_MBL_**
1.	***Escherichia coli***	AK-69	KX231909	NDM-7	Positive	Present	OXA-1, CMY-1	38, 6, 4	FIA, FIC, F, K	Class 1	Complete	Present
2.		AK-70	KX231910	NDM-5	Positive	Present	OXA-1	154, 38, 4	FIA, FIC, F, K	Class 1	Truncated	Present
3.		AK-71	KX231911	NDM-5	Positive	Present	CMY-1	66, 38, 6, 4	FIA, FIB, F, K	Class 1	Complete	Present
4.		AK-72	KX231912	NDM-5	Positive	Present	OXA-1	154, 66, 38, 6	FIA, FIC, F, K	Class 1	Complete	Present
5.		AK-74	KX231914	NDM-5	Positive	Present	CMY-149	66, 38, 6	I, F, K	Class 1	Complete	Present
6.		AK-76	KX231915	NDM-5	Positive	Present	OXA-1	154, 38	FIA, F, K	Class 1	Complete	Present
7.		AK-77	KX231916	NDM-5	Positive	Present	OXA-1, CMY-149	66, 38, 6, 4	FIA, FIB, I, B/O, K	Class 1	Complete	Present
8.		AK-79	KX231918	NDM-5	Positive	Present	OXA-1, CMY-1	38	FIA, FIB, F, K	Class 1	Complete	Present
9.		AK-80	KX231919	NDM-5	Positive	Present	OXA-1	38, 2	FIA, FIB, F, K	Class 1	Complete	Present
10.		AK-81	KX231920	NDM-5	Positive	Present	OXA-1, CMY-1	38, 6, 4	I, F, K	Class 1	Truncated	Present
11.		AK-83	KX231922	NDM-7	Positive	Present	OXA-1, SHV-1	38, 25	FIA, FIB, F, K	Class 1	Complete	Present
12.		AK-86	KX231925	NDM-5	Positive	Present	OXA-1, CMY-1	38, 6	FIA, F, K	Class 1	Complete	Present
13.		AK-87	KX231926	NDM-5	Positive	Present	OXA-1	38, 6, 4	FIA, F, K	Class 1	Complete	Present
14.		AK-88	KX231927	NDM-5	Positive	Present	OXA-1, OXA-9	154, 66	FIA, F, K	Class 1	Complete	Present
15.		AK-90	KX231929	NDM-5	Positive	Present	OXA-1	38, 4	FIA, F, K	ND	Complete	Present
16.		AK-91	KX231930	NDM-5	Positive	Present	OXA-1	154, 66	FIA, F, I, K	Class 1	Complete	Present
17.		AK-105	KX999132	NDM-5	Positive	Present	OXA-1, OXA-9, CMY-1	154, 66, 38	HI1, Y, FIA, FIB, F, K	Class 1	Truncated	Present
18.		AK-107	KX999134	NDM-4	Positive	Present	OXA-1, OXA-9, SHV-1	66, 38	I, FIA, FIB, F, FIIA	Class 1	Complete	Present
19.		AK-109	KX999136	NDM-5	Positive	Present	CMY-149	38, 6, 4	I, F, K	Class 1	Complete	Present
20.		AK-116	KX999143	NDM-1	Positive	Present	SHV-2	154	FIA, FIC	Class 1	Complete	Present
21.	***Klebsiella pneumoniae***	AK-66	KX231906	NDM-1	Positive	Present	OXA-1, OXA-9, CMY-1	38	FIIA, FIC.	Class 1	Complete	Present
22.		AK-78	KX231917	NDM-1	Positive	Present	OXA-1	148	FIIA	Class 1	Truncated	Present
23.		AK-85	KX231924	NDM-1	Positive	Present	OXA-9, CMY-145	38, 6, 4	FIA, F, K	Class 1	Complete	Present
24.		AK-89	KX231928	NDM-1	Positive	Present	OXA-1	38	FIIA	Class 1	Complete	Present
25.		AK-94	KX999121	NDM-1	Positive	Present	CMY-145, SHV-1	154, 66, 38	Y, FIA, K, FIIA	Class 1	Complete	Present
26.		AK-97	KX999124	NDM-4	Positive	Present	OXA-1, OXA-9	154, 66, 38, 6, 4	P, FIC, FIA, FIB, F, K	Class 1	Complete	Present
27.		AK-98	KX999125	NDM-4	Positive	Present	OXA-1, OXA-9, CMY-1, SHV-1	38, 6	K, FIIA	Class 1	Truncated	Present
28.		AK-99	KX999126	NDM-4	Positive	Present	OXA-1, OXA-9, SHV-2	38, 6	K, FIIA	Class 1	Truncated	Present
29.		AK-101	KX999128	NDM-4	Positive	Present	OXA-1, OXA-9, CMY-145	154, 66, 38, 6, 4	P, FIC, FIA, FIB, F, K	Class 1	Complete	Present
30.		AK-102	KX999129	NDM-5	Positive	Present	OXA-1, OXA-9, CMY-4	154, 66, 38,	FIIA	Class 1	Complete	Present
31.		AK-103	KX999130	NDM-4	Positive	Present	OXA-1, OXA-9	66	FIC, K	ND	Complete	Present
32.		AK-104	KX999131	NDM-4	Positive	Present	OXA-1, OXA-9, CMY-4, SHV-1	38, 6, 4	P, FIC, K, FIIA	Class 1	Complete	Present
33.		AK-106	KX999133	NDM-4	Positive	Present	OXA-1, OXA-9, SHV-2	38, 6, 4	K	Class 1	Complete	Present
34.		AK-110	KX999137	NDM-4	Positive	Present	OXA-1, OXA-9, CMY-145	38, 6, 4	K, FIIA	Class 1	Truncated	Present
35.		AK-111	KX999138	NDM-4	Positive	Present	OXA-1, OXA-9	38, 6, 4	K, FIIA	Class 1	Complete	Present
36.		AK-112	KX999139	NDM-1	Positive	Present	OXA-1	66, 38	K, FIIA	Class 1	Truncated	Present
37.		AK-114	KX999141	NDM-4	Positive	Present	OXA-1, OXA-9, SHV-1	66, 38	K, FIIA	Class 1	Complete	Present
38.		AK-115	KX999142	NDM-4	Positive	Present	OXA-1, OXA-9	38, 6	Y, FIA, FIB, F, K, FIIA	Class 1	Complete	Present
39.	***Citrobacter freundii***	AK-82	KX231921	NDM-4	Positive	Present	OXA-9, SHV-1, CMY-149	38	N, F, K	Class 1	Complete	Present
40.		AK-113	KX999140	NDM-1	Positive	Present	OXA-1, SHV-2, CMY-149	66	FIC, K	Class 1	Truncated	Present
41.	***Citrobacter braakii***	AK-84	KX231923	NDM-4	Positive	Present	OXA-1, CMY-145	38	F	Class 1	Complete	Present
42.	***Klebsiella oxytoca***	AK-100	KX999127	NDM-4	Positive	Present	OXA-1, OXA-9	154, 66, 38	I, Y, FIA, F, K, FIIA	Class 1	Complete	Present
43.	***Enterobacter cloacae***	AK-108	KX999135	NDM-4	Positive	Present	OXA-1, OXA-9, CMY-149	66,38	FIA, FIB	Class 1	Truncated	Present
44.	***Enterobacter aerogenes***	AK-67	KX231907	NDM-1	Positive	Present	OXA-1, SHV-2	154, 38, 6, 4	N, FIIA, FIC, K	Class 1	Truncated	Present

* *These features were also found on transconjugants*.

### Carbapenemase production

All 44 NDM- producing enterobacteriaceae isolates were found positive for Carba-NP test, indicating the production of a carbapenemase as shown in Table 1.

### Detection of antibiotic resistance markers

PCR amplification and sequencing confirmed that all isolates harbored *bla*_NDM_ of which NDM-1 (9; 20.45%), NDM-4 (16; 36.36%), NDM-5 (17; 38.64%), and NDM-7 (2; 4.55%) were found to be prevalent. Sequences were submitted to NCBI database (Table 1). Further *bla*_CMY_ was detected in 20 isolates (08; *bla*_CMY−1_, 02; *bla*_CMY−4_, 05; *bla*_CMY−145_, and 05; *bla*_CMY−149_) whereas, *bla*_OXA−1_ was detected in 37 isolates, and *bla*_OXA−9_ was found in 20 isolates. Moreover, 07 *bla*_SHV−1_ and 05 *bla*_SHV−2_ were also found in this study (Table 1). However, *bla*_TEM_, *bla*_VIM_, *bla*_IMP_, and *bla*_KPC_ were not detected in any of these isolates. Conjugation experiment, further confirmed the presence of these resistance markers on plasmid in each isolate.

### Conjugation

The plasmidic location of resistant markers was determined by conjugation, using an azide-resistant *E. coli* J53 strain as the recipient [12]. Transconjugants were obtained at the frequencies of 10^−3^ to 10^−5^ cells, showing that plasmid from the donors (*E. coli, K. pneumoniae, C. freundii, C. braakii, K. oxytoca, E. cloacae, E. aerogenes*), were found stable in *E. coli* J53.

### Replicon typing

These studied NDM producing isolates contained detectable plasmid size (154kb, 66kb, 38kb, 6kb, and 4kb) as shown in Table 1. Number of plasmids were found in the isolates, 1(*n* = 09), 2(*n* = 14), 3(*n* = 15), 4(*n* = 04), 5(*n* = 02). PBRT method identified 12 of 18 replicons types in our study while, IncHI2, IncL/M, IncW, IncT, IncA/C, and IncX were not detected in this study. IncFIA (*n* = 24), IncFIC (*n* = 11), IncF (*n* = 25), IncK (*n* = 36), IncFIB (*n* = 11), IncB/O (*n* = 01), IncHI1 (*n* = 01), IncP (*n* = 03), IncY (*n* = 04), IncFIIA (*n* = 16), IncI1 (*n* = 07), and IncN (*n* = 02), replicon types were predominant in the present study and IncFIA, IncFIC, IncF, IncK, and IncFIB were found to be the most frequent types in this study.

### Integron analysis

The transconjugants of all isolates harbored plasmid carrying class 1 integron, except two isolates (AK-90 and AK-103) which were confirmed by PCR amplification of 5′/3′ CS, *Int*I, and *Sul*I genes. We further confirmed that no resistant marker was present in the integron cassette as shown by a PCR using amplicon of 5′/3′ CS as template.

### Genetic relatedness of the carbapenem resistant NDM producing enterobacteriaceae isolates

**Figure 1:**
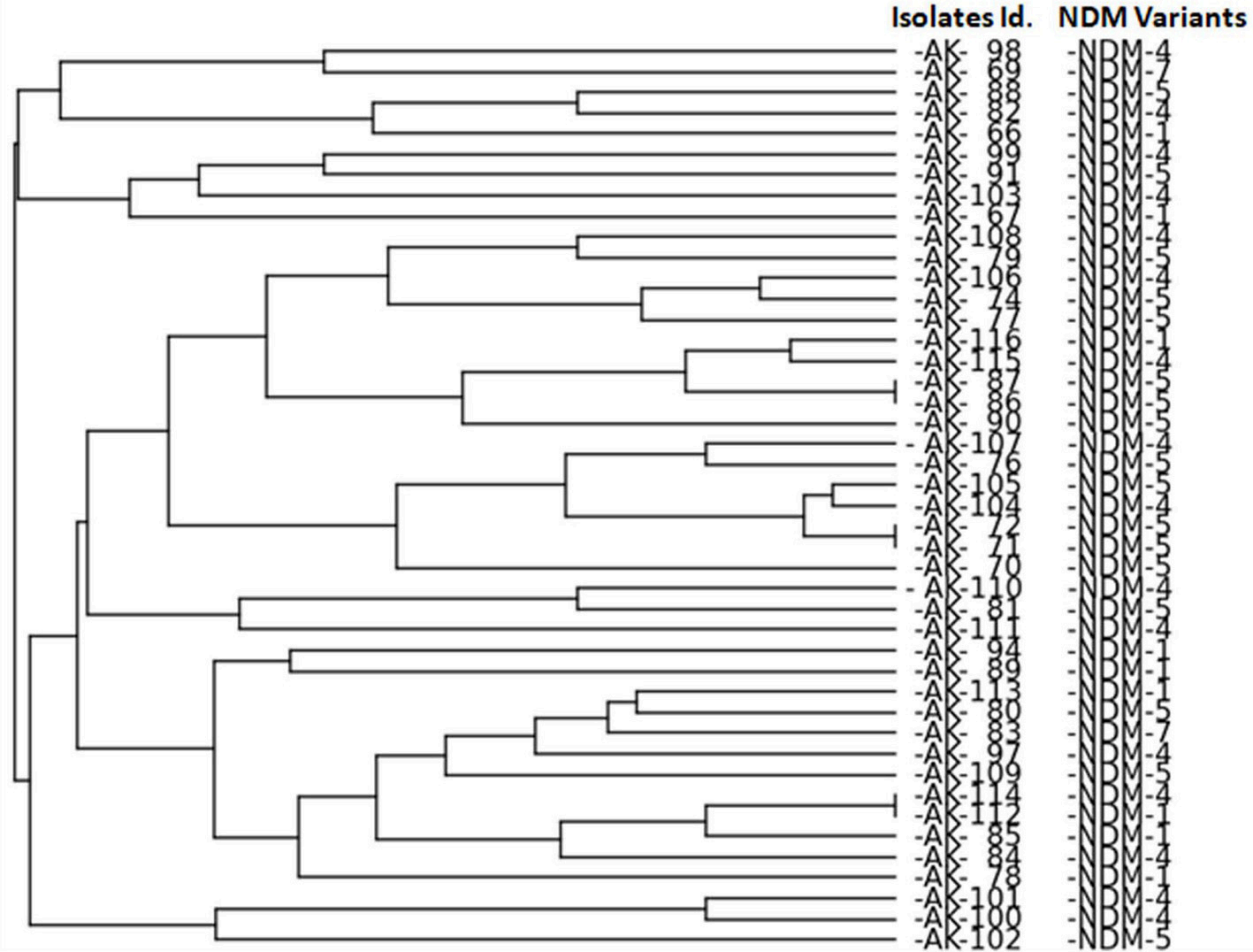
ERIC PCR analysis of NDM producing isolates. Bio-Red Gel Doc system was used to analyze the bands by PyElph version 1.4 Software generate a dendrogram by the unweighted pair group method using arithmetic averages (UPGMA) clustering. Generated dendogram showing genetic relationship among NDM producing isolates.

ERIC-PCR analysis revealed no clonal relatedness among isolates except for the isolates of *K. pneumoniae* (AK-86 with AK-87, AK-71 with AK-72 and AK-112 with AK-114) as shown in Figure 1.

### Genetic environment of the *bla*_NDM_ gene

**Figure 2:**
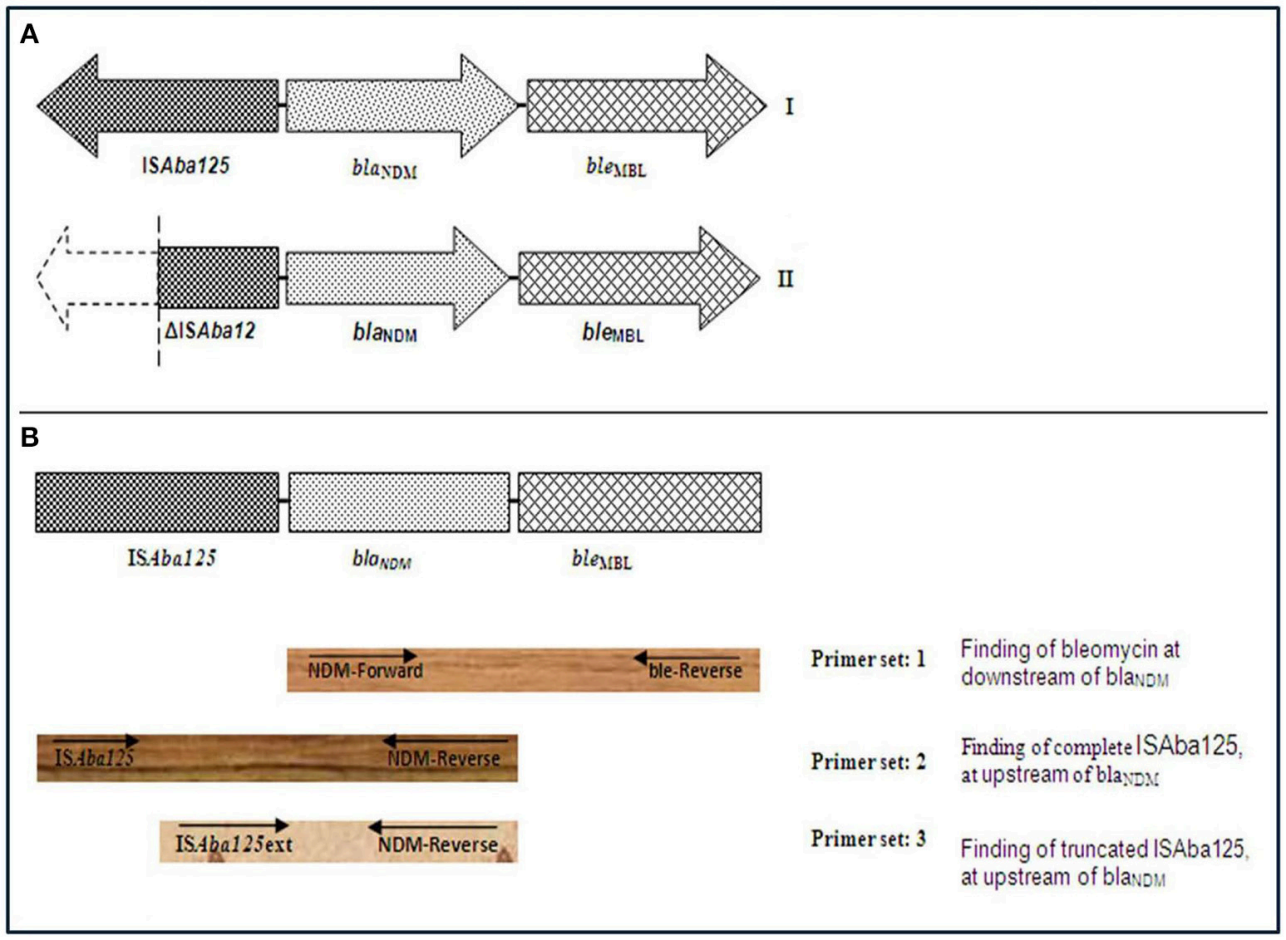
**(A)** A schematic representation of genetic elements surrounding *bla*_NDM_. (I) In AK-69, AK-71, AK-72, AK-74, AK-76, AK-77, AK-79, AK-80, AK-83, AK-86, AK-87, AK-88, AK-90, AK-91, AK-107, AK-109, AK-116, AK-66, AK-85, AK-89, AK-94, AK-97, AK-101, AK-102, AK-103, AK-104, AK-106, AK-111, AK-114, AK-115, AK-84, AK-82, and AK-100, complete element of IS*Aba125* at upstream and bleomycin gene at downstream to *bla*_NDM_ was found. (II) In AK-70, AK-81, AK-105, AK-78, AK-98, AK-99, AK-110, AK-112, AK-113, AK-108, and AK-67, truncated IS*Aba125* at upstream and bleomycin gene at downstream to *bla*_NDM_ was found. **(B)** A schematic representation for PCR-based genetic environment analysis of *bla*_NDM_. Arrow indicates the position of primer (use of primers as described in reference Poirel et al., 2011).

PCR based genetic environment analysis of *bla*_NDM_ gene was performed and *ble*_MBL_ was found at downstream of *bla*_NDM_ variants in all isolates (Figure 2). A complete IS*Aba125* sequence was found at upstream of *bla*_NDM_ in one *bla*_NDM−1_ (AK-116), one *bla*_NDM−4_ (AK-107), 13 *bla*_NDM−5_ (AK-71, AK-72, AK-74, AK-76, AK-77, AK-79, AK-80, AK-86, AK-87, AK-88, AK-90, AK-91, and AK-109,) and two NDM-7 producing *E. coli* (AK-69 and AK-83). Further, complete IS*Aba125* was amplified in 4 isolates of NDM-1(AK-66, AK-85, AK-89, and AK-94), eight isolates of NDM-4 (AK-97, AK-101, AK-103 AK-104, AK-106, AK-111, AK-114, and AK-115) and one (AK-102) NDM-5 producing *K. pneumoniae* (Figure 2). A complete IS*Aba125* was amplified in three isolates of NDM-4 producing *C. freundii, C. braakii*, and *K. oxytoca*, respectively (AK-84, AK-82, and AK-100). However, truncated IS*Aba125* was detected in three isolates of NDM-5 producing *E. coli* (AK-70, AK-81, and AK-105). Moreover, 2; NDM-1 (AK-78, AK-112), 3; NDM-4 (AK-98, AK-99, and AK-110), producing *K. pneumoniae* and one NDM-1 (AK-113) producing *C. freundii, one* NDM-4 (AK-108) producing *E. cloacae* and one (AK-67) NDM-1 producing *E. aerogenes* had truncated IS*Aba125* at upstream of *bla*_NDM_ (Table 1, Figure 2).

## Discussion

**Figure 3:**
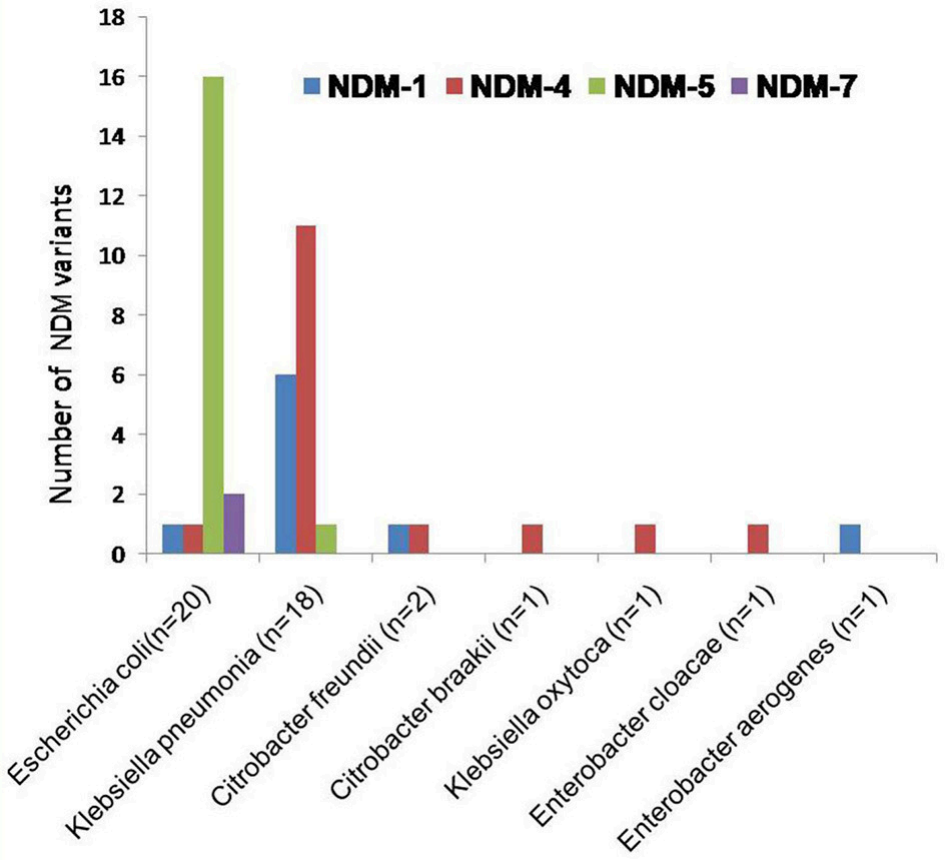
The clustered bar graph presents the number of NDM variants (each is represented by its own bar) distributed among NDM-producing enterobacteriaceae collected from NICU. The horizontal axis represents the NDM-producing enterobacteriaceae while the vertical axis represents the number of NDM variants.

Emergence of NDM-producing enterobacteriaceae has become a globally serious concern. NDM producers led to limited therapeutic options hence it has become a threat to public health. Epidemiological investigation and surveillance of NDMs are of importance to clinical infection control. This study revealed outbreak of multiple variants of *bla*_NDM_ (9; *bla*_NDM−1_, 16; *bla*_NDM−4_, 17; *bla*_NDM−5_, and 2; *bla*_NDM−7_) in clinically important bacteria (20 *E. coli*, 18 *K. pneumoniae*, 02 *C. freundii*, 01 *C. braakii*, 01 *K. oxytoca*, 01 *E. cloacae*, 01 *E. aerogenes*), as shown in Figure 3.

In *E. coli* the predominant NDM variant was found to be *bla*_NDM−1_, followed by *bla*_NDM−4_, *bla*_NDM−5_, and *bla*_NDM−7_ (Figure 3). Although this is not first description of these NDM variants being produced by *E. coli* (Zhang et al., 2013; Qin et al., 2016; Zhu et al., 2016; Pál et al., 2017). Moreover, in these strains existence of NDM and its variants, with CMY, OXA, SHV, and VIM variants and other resistant determinants are documented. Of 20 NDM producing *E. coli*, one NDM-1 isolate (AK-116) was coexisting with *bla*_SHV−2_ and one NDM-4 isolate (AK-107) coexisting with *bla*_OXA−1_, *bla*_OXA−9_, and *bla*_SHV−1_. Further, two isolates of NDM-7 (AK-69, AK-83) were associated with *bla*_OXA−1_, *bla*_SHV−1_, *bla*_CMY−1_, and 16 isolates of *bla*_NDM−5_ were linked to *bla*_OXA−1_, *bla*_OXA−9_, *bla*_SHV−1_, *bla*_CMY−1_, or *bla*_CMY1−49_ in different combinations.

The most prevalent NDM variants in *K. pneumoniae* is *bla*_NDM−4_, followed by *bla*_NDM−5_ and *bla*_NDM−1_ (Figure 3). It has also been shown in earlier studies in *Klebsiella pneumonia* (Khalifa et al., 2016; Petersen-Morfin et al., 2017). Of 18 NDM producing *K. pneumoniae*, 6 were NDM-1 isolates, coexisting with *bla*_OXA−1_, *bla*_OXA−9_, *bla*_SHV−1_, *bla*_CMY−1_, and *bla*_CMY−145._ Further, 11 NDM-4 isolates were found associated with *bla*_OXA−1_, *bla*_OXA−9_, *bla*_SHV−1_, *bla*_SHV−2_, *bla*_CMY−1_, *bla*_CMY−149_, and *bla*_OXA−1_, *bla*_OXA−9_, *bla*_CMY−4_ in association with *bla*_NDM−5._

*Citrobacter* species are rare opportunistic nosocomial pathogens (Ryan and Ray, 2004). It normally causes urinary tract infections, blood stream infections, intra-abdominal sepsis, brain abscesses, pneumonia, and other neonatal infection (Pepperell et al., 2002) such as meningitis, neonatal sepsis, joint infection, or general bacteremia (Doran, 1999). The principal NDM variant found in *C. freundii* was *bla*_NDM−1_ which was followed by *bla*_NDM−4._ It is a first report of NDM-4 producing *C. freundii*, (AK-82) co-associated with *bla*_OXA−9_, *bla*_SHV−1_, and *bla*_CMY−149_. Further, *C. freundii* (AK-113) was also found to have *bla*_OXA−1_, *bla*_SHV−2_, and *bla*_CMY−149_ in association with *bla*_NDM−1_.

Moreover, for the first time NDM-4 producing *C. braakii* (AK-84), *K. oxytoca* (AK-100), and *E. cloacae* (AK-108) were identified in association with *bla*_OXA−1_ and *bla*_CMY−145_, *bla*_OXA−1_ and *bla*_OXA−9_ and, *bla*_OXA−1_, *bla*_OXA−9_, and *bla*_CMY−149_, respectively.

We have also identified NDM-1 producing *E. aerogenes* co-associated with *bla*_OXA−1_ and *bla*_SHV−2_ in AK-67. NDM-1 producing *C. braakii*, in Pakistan (Pesesky et al., 2015), NDM-1 producing *K. oxytoca* in China (Wang et al., 2017), NDM-1 producing *E. cloacae* in Turkey (Haciseyitoglu et al., 2017) and Coratia (Petrosillo et al., 2016), have been reported in earlier studies.

The transconjugants were stable and carried all the resistant determinants from donor. Moreover, the presence of class 1 integron in all isolates except AK-90 and AK-103, suggests that the resistant markers can competently exchange among species leading to its spread in the hospital (Martinez-Freijo et al., 1998). Presence of resistance genes on plasmids of varying sizes (4–154 kb) were identified in this study. Previous studies have proved to have these resistance genes on plasmid of size 7–200 kb (Mshana et al., 2009). The replicon typing revealed varying replicon types (IncFIA, IncFIB, IncFIC, IncFIIA, IncF, IncN, IncK, IncB/O, IncHI1, IncY, IncI1, and IncP). In previous studies, *bla*_NDM_ gene was shown to be associated with plasmid type (IncFIA IncFIB) (Gamal et al., 2016), (IncX3) (Zhang et al., 2016), (IncFIC, IncF, and IncK) (Ahmad et al., 2017a), (IncB/O) (An et al., 2016), (IncHI1, IncN, and IncFIIA) (Sartor et al., 2014), (IncY, IncA/C IncI1) (Kapmaz et al., 2016). Moreover, first time we have identified three NDM-4 producing *Klebsiella pnemoniae* with incompatibility group IncP in AK-97, AK-101, and AK-104 strains.

Complete IS*Aba125* sequence was observed at upstream of *bla*_NDM_ in most of the isolates implies that this factor may play a main role in horizontal gene transfer of the *bla*_NDM_ among enterobacteriaceae members (Poirel et al., 2011). In all *bla*_NDM_ variants, *ble*_MBL_ was found at it downstream. The occurrence of *ble*_MBL_, associated with *bla*_NDM_ gene, suggests that they might have mobilized simultaneously from same progenitor and is thought to protect *bla*_NDM_ (Dortet et al., 2012)_._ These results suggest that the plasmids encoding for carbapenem resistant NDM variants can easily spread among the enterobacteriaceae isolates. These results are in conformity with previous reports that clarified the horizontal transfer of plasmids encoding for carbapenemases among enterobacteriaceae including *K. pneumoniae* (Dortet et al., 2014; Jin et al., 2015).

## Conclusions

Carbapenem resistance among enterobacteriaceae has been considered as one of the most significant menaces to the global healthcare, and the prevalence of NDM variants in enterobacteriaceae has further increased the threat. Therefore, the early detection of the *bla*_NDM_ possessing enterobacteriaceae isolates with any decreased sensitivity to the carbapenems is crucial for the choice of the most appropriate antibiotic therapy and the application of efficient infection control measures. The emergence of such resistance patterns may be reduced by the restricted implementation of antibiotics, especially for carbapenems and cephalosporins. Moreover, a strong infection control management in the hospital is necessary to check such infection.

## Author contributions

NA: performed experiments, wrote draft manuscript; SK: performed experiments; SA: provided samples, and interpreted clinical data; AK: designed study and checked draft manuscript.

### Conflict of interest statement

The authors declare that the research was conducted in the absence of any commercial or financial relationships that could be construed as a potential conflict of interest. The handling Editor declared a past co-authorship with one of the authors AK.
