# Supplementary material for: Occurrence of blaNDM Variants Among Enterobacteriaceae From a Neonatal Intensive Care Unit in a Northern India Hospital
Source: Front Microbiol. 2018 Mar 7;9:407. doi: 10.3389/fmicb.2018.00407 (PMC5845868; doi:10.3389/fmicb.2018.00407)
Supplement: Supplementary file 1 [file Table1.DOCX]

**Supplementary Table S1.** Minimum Inhibitory Concentrations (MICs) values for NDM-producing enterobacteriaceae isolated from NICU setting

| **S.No** | **Isolate Id** | **MIC (µg ml^-1^)** | | | | | | | | | |
| --- | --- | --- | --- | --- | --- | --- | --- | --- | --- | --- | --- |
|  |  | **MRP** | **IMP** | **CTX** | **CAZ** | **CX** | **CXM** | **CIP** | **FEP** | **GEN** | **ATM** |
| 1. | AK-66 | 1024 | >1024 | 2048 | 2048 | 2048 | >1048 | 512 | 2048 | 1024 | >1024 |
| 2. | AK-67 | 1024 | 1024 | >1024 | 2048 | >2048 | 4096 | >512 | >2048 | 1024 | 1024 |
| 3. | AK-69 | 1024 | 2048 | 4096 | >2048 | 4096 | >2048 | 1024 | 2048 | >512 | 1024 |
| 4. | AK-70 | 1024 | >1024 | 4096 | >2048 | 4096 | 2048 | 1024 | 4096 | 1024 | >512 |
| 5. | AK-71 | >1024 | 2048 | 4096 | 4096 | >2048 | 4096 | 512 | 2048 | 1024 | 1024 |
| 6. | AK-72 | 512 | 1024 | >1024 | 1024 | 2048 | >1024 | 1024 | 4096 | >512 | 1024 |
| 7. | AK-74 | 1024 | 1024 | 2048 | >1024 | >2048 | 2048 | 1024 | 2048 | >512 | 2048 |
| 8. | AK-76 | 1024 | >1024 | 2048 | 2048 | >1024 | 2048 | 512 | 4096 | 1024 | 1024 |
| 9. | AK-77 | 1024 | 1024 | >1024 | >1024 | 4096 | >2048 | 1024 | 2048 | 1024 | 2048 |
| 10. | AK-78 | >1024 | 2048 | 4096 | 2048 | >2048 | 4096 | >512 | 2048 | 512 | 1024 |
| 11. | AK-79 | 1024 | 1024 | 2048 | >1024 | >1024 | 2048 | 1024 | 4096 | 1024 | 2048 |
| 12. | AK-80 | 1024 | >1024 | 2048 | >2048 | 2048 | 4096 | 512 | 2048 | >512 | 1024 |
| 13. | AK-81 | 1024 | 1024 | >1024 | 2048 | >2048 | 4096 | 1024 | 4096 | 512 | 2048 |
| 14. | AK-82 | >1024 | 2048 | 4096 | >2048 | 4096 | >2048 | >512 | 2048 | 1024 | >1024 |
| 15. | AK-83 | 1024 | 1024 | >1024 | 2048 | 4096 | >2048 | 512 | 2048 | >512 | 2048 |
| 16. | AK-84 | 1024 | 2048 | >2048 | 2048 | >2048 | 4096 | 1024 | >2048 | 512 | >1024 |
| 17. | AK-85 | 1024 | 1024 | 2048 | >1024 | 2048 | >1024 | 512 | 2048 | 1024 | 2048 |
| 18. | AK-86 | 512 | 1024 | >1024 | 2048 | 2048 | >2048 | 1024 | 2048 | >512 | 2048 |
| 19. | AK-87 | 1024 | 1024 | 2048 | >2048 | 4096 | 2048 | 512 | >2048 | 1024 | >2048 |
| 20. | AK-88 | 1024 | >1024 | 2048 | 4096 | >2048 | 2048 | 1024 | 2048 | >512 | 2048 |
| 21. | AK-89 | 512 | >512 | 1024 | 1024 | 1024 | >1024 | 512 | 1024 | 512 | >1024 |
| 22. | AK-90 | 512 | 1024 | 2048 | >1024 | 2048 | 1024 | 512 | 1024 | 512 | 1024 |
| 23. | AK-91 | >512 | 1024 | >1024 | 2048 | >1024 | 2048 | >512 | 2048 | 512 | 1024 |
| 24. | AK-94 | 256 | 512 | 1024 | >1024 | 2048 | >1024 | 128 | 2048 | >256 | 1024 |
| 25. | AK-97 | 1024 | 1024 | 2048 | >1024 | 2048 | 4096 | >512 | >1024 | >512 | >1024 |
| 26. | AK-98 | 1024 | >1024 | 2048 | >1024 | 2048 | 4096 | 1024 | 2048 | 512 | 1024 |
| 27. | AK-99 | >512 | 1024 | >1024 | 2048 | 2048 | >1024 | 512 | >1048 | >512 | 1024 |
| 28. | AK-100 | 1024 | >1024 | 2048 | >2048 | 4096 | >1024 | >512 | 2048 | 1024 | 1024 |
| 29. | AK-101 | 512 | 1024 | >1024 | 2048 | >1024 | 2048 | 512 | >1024 | 512 | >512 |
| 30. | AK-102 | 1024 | >1024 | 2048 | 4096 | >2048 | 4096 | >512 | 2048 | 512 | 1024 |
| 31. | AK-103 | 1024 | 1024 | 2048 | >1024 | 2048 | >2048 | 512 | 2048 | >512 | 1024 |
| 32. | AK-104 | 512 | 1024 | >2048 | 2048 | 4096 | 4096 | >512 | >2048 | 512 | 1024 |
| 33. | AK-105 | 256 | 512 | 1024 | >1024 | 1024 | 2048 | 128 | 2048 | >256 | 512 |
| 34. | AK-106 | 1024 | 1024 | >1024 | 2048 | >1024 | 2048 | 512 | 2048 | 512 | 1024 |
| 35. | AK-107 | >1024 | 2048 | >2048 | 4096 | 4096 | >2048 | 1024 | 4096 | >512 | 1024 |
| 36. | AK-108 | 512 | >512 | 1024 | 1024 | 2048 | >1024 | 512 | 1024 | >256 | 512 |
| 37. | AK-109 | 1024 | >1024 | 2048 | >2048 | 4096 | 2048 | 512 | >1024 | >512 | 1024 |
| 38. | AK-110 | >1024 | 2048 | 4096 | >2048 | >2048 | 4096 | 1024 | >2048 | 512 | 1024 |
| 39.. | AK-111 | 1024 | >1024 | >2048 | 4096 | 2048 | >2048 | >512 | 4096 | 512 | 512 |
| 40. | AK-112 | 1024 | 1024 | >1024 | 2048 | >1024 | >2048 | 1024 | >2048 | >512 | 512 |
| 41. | AK-113 | 1024 | 1024 | 2048 | >1024 | >2048 | 4096 | >512 | 4096 | 512 | 1024 |
| 42. | AK-114 | 1024 | >1024 | 4096 | 2048 | >2048 | 4096 | >512 | >2048 | 512 | 1024 |
| 43. | AK-115 | 512 | 1024 | 2048 | 4096 | >1024 | 2048 | 1024 | 4096 | >512 | 1024 |
| 44. | AK-116 | 512 | >512 | 2048 | 1024 | >2048 | 4096 | >512 | >2048 | 512 | 1024 |

MRM: meropenem, IPM: imipenem, CTX: cefotaxime, CAZ: ceftazidime, CX: cefoxitin CXM: cefuroxime, CIP: ciprofloxacin, FEP: cefepime, GEN: gentamicin, ATM: aztreonam
